# Lipid Profile Alterations in Pediatric Patients with Vitamin D Deficiency

**DOI:** 10.3390/children12050546

**Published:** 2025-04-24

**Authors:** Jasmina Katanić, Dejan Dobrijević

**Affiliations:** 1 Institute for Children and Youth Health Care of Vojvodina, 21000 Novi Sad, Serbia; jasmina.katanic@mf.uns.ac.rs; 2 Faculty of Medicine, University of Novi Sad, 21000 Novi Sad, Serbia

**Keywords:** vitamin D deficiency, lipid profile, dyslipidemia, pediatric population, vitamin D supplementation, serum lipids

## Abstract

**Background/Objectives**: Vitamin D deficiency in children has been linked to various metabolic disturbances, including dyslipidemia, which contributes to cardiovascular risk. This study aims to investigate the relationship between vitamin D levels and lipid profiles in children. **Methods**: A cohort of 332 children with either normal vitamin D or diagnosed vitamin D deficiency was recruited. Serum vitamin D levels were measured, and lipid profiles, including total cholesterol, low-density lipoproteins (LDLs), high-density lipoproteins (HDLs), and triacylglycerols (TAGs), were assessed. The data were analyzed using statistical methods. **Results**: This study found that children with higher serum vitamin D concentrations had significantly lower TAG (*p* = 0.033) and very-low-density lipoprotein (VLDL) (*p* = 0.038) levels and higher HDL levels (*p* = 0.042), indicating a more favorable lipid profile compared to those with lower vitamin D levels. **Conclusions**: This study demonstrates that vitamin D deficiency can be associated with dyslipidemia in children. These findings suggest that vitamin D supplementation may be an effective strategy for managing dyslipidemia and reducing cardiovascular risk in pediatric populations. Further research is needed to determine the long-term effects and optimal dosing of vitamin D in this context.

## 1. Introduction

The timely diagnosis of lipid status disorders in the form of hyperlipidemia is crucial for the prevention of cardiovascular diseases, as lipid levels in childhood directly impact the lipid profile of patients in adulthood [1]. In support of this, it has been observed that low levels of vitamin D may potentially be associated with the lipoprotein profile, thus increasing the risk of cardiovascular diseases and other secondary etiological factors in the pathogenesis of major non-communicable diseases, such as elevated blood pressure or excessive body weight [2]. Therefore, monitoring this condition in the pediatric patient population is of great importance to prevent more severe complications and pathological conditions due to neglect of health, with the goal of improving therapeutic approaches and quality of life in later years.

Vitamin D is a fat-soluble hormone that is naturally synthesized in the body through subcutaneous synthesis after exposure to sunlight and plays a crucial role in various biological functions [3]. It is one of the essential hormones for normal growth, development, and the survival of the human organism, especially in children. The primary role of vitamin D is to maintain serum calcium levels within the physiological range for the normal functioning of the nervous system, as well as for bone growth and maintaining bone density. Vitamin D is necessary for the effective use of calcium in the body [4]. The importance of vitamin D has also been demonstrated in the regulation of certain cardiometabolic functions and pathological conditions that arise due to disruptions in these functions, including insulin resistance, type 2 diabetes, hypertension, and dyslipidemia. Dyslipidemia is one of the main risk factors for the development of cardiovascular diseases (CVD), and some studies have shown that serum vitamin D levels correlate with a more favorable lipid profile compared to deficient levels [5].

Although several mechanisms have been proposed to explain the effects of vitamin D on the lipid profile, the impact of this vitamin in this regard is still not fully clarified. Proposed mechanisms suggest that vitamin D may directly affect the serum lipid profile, including TAG, total cholesterol, and low-density lipoproteins (LDLs), by increasing bile salt production and reducing lecithin-cholesterol acyltransferase activity, and it may indirectly affect the profile through its impact on calcium absorption, resulting in reduced fat absorption and the increased synthesis of liver bile acids from cholesterol [3].

In recent years, there has been a growing trend in vitamin D deficiencies. The primary source of vitamin D is exposure to sunlight, and protein-rich foods also contain certain amounts of vitamin D. The reference nutrient intake (RNI) for vitamin D in the pediatric population is 10 µg/day or 400 IU. According to the German Nutrition Society and national pediatric recommendations, supplementation is advised from birth to the first year of life and during winter months in the second year. Beyond this period, endogenous synthesis from sunlight becomes the primary source [6]. However, dietary intake alone may not meet an individual’s vitamin D needs [7].

The aim of the study was to investigate the existence of differences in the lipid profile depending on the levels of vitamin D within a pediatric patient group with an existing diagnosis of hypovitaminosis D.

## 2. Materials and Methods

A retrospective study was conducted at the Department of Laboratory Diagnostics of the Institute for Children and Youth Health Care of Vojvodina in Novi Sad. The study included a total of 332 pediatric patients, treated between December 2023 and January 2025. The parameters investigated were collected by reviewing the Laboratory Information System of the Department of Laboratory Diagnostics of the Institute and included the following: vitamin D levels, triacylglycerols (TAG), cholesterol, high-density lipoproteins (HDLs), low-density lipoproteins (LDLs), very-low-density lipoproteins (VLDLs), gender, and age. The body mass index (BMI) was also calculated, which was considered when interpreting the results. The exclusion criteria for the study were as follows: chronic diseases, malignancies, and patients with acute infections.

For the reference range of vitamin D levels, the following values were used: physiological values ≥ 50 nmol/L, low values 30–49 nmol/L, and very low values < 30 nmol/L [8]. Lipid levels were interpreted according to the NHLBI Expert Panel guidelines [1]. The reference values in mmol/L are as follows: Total cholesterol is acceptable if <4.40, borderline if 4.40–5.15, and high if ≥5.17; LDL is acceptable if <2.84, borderline if 2.84–3.34, and high if ≥3.36; HDL is acceptable if >1.17, borderline if 1.03–1.17, and low if <1.03; triglycerides are acceptable if <0.85 for children under 10 years and <1.02 for children ≥ 10 years; estimated VLDL is considered acceptable if <0.85. Biochemical parameters were determined using automatic biochemical analyzer D×C 700 AU (Beckman Coulter, Brea, CA, USA), while vitamin D levels were determined using automatic immunoassay analyzer Access 2 (Beckman Coulter, Brea, CA, USA). For all analyses, blood serum samples were used, specifically vacuum tubes with clot activators (Becton Dickinson, Franklin Lakes, NJ, USA).

Data analysis was performed using descriptive and inferential statistical methods. The chi-square test was used to compare categorical variables (gender). Differences between numerical variables were determined using ANCOVA, ANOVA, and Kruskal–Wallis tests. The ANCOVA statistical method was used to analyze covariates (in this case, BMI) that could have influenced the dependent variable, i.e., the research outcome (vitamin D levels). A statistically significant difference was considered if the *p*-value was <0.05. Data processing was carried out using the SPSS statistical software package, version 26.0 (IBM, Armonk, NY, USA).

The study was approved by the Ethics Committee of the Institute for Children and Youth Health Care of Vojvodina in Novi Sad (20 March 2025, no. 1641).

## 3. Results

The study included 332 patients, consisting of 190 boys and 142 girls, with either physiological vitamin D levels (≥50 nmol/L) or hypovitaminosis D. The average age of the entire study group was 12.1 years, with boys having an average age of 13.2 years and girls 11.6 years. Regarding sex as a categorical variable, the distribution was balanced without statistically significant differences (*p* > 0.05).

Table 1 presents the differences in lipid status parameters depending on the measured serum levels of vitamin D. It was determined that there were statistically significant differences regarding TAG, HDL, and VLDL, while for CHOL and LDL, no statistically significant differences were observed.

Based on the analyzed values of serum vitamin D levels retrieved from the Laboratory Information System of the Institute, the arithmetic mean of vitamin D levels for all patients was calculated and categorized according to the reference ranges (Table 1). According to the measured values, a statistically significant difference in vitamin D levels was found between the population groups of patients (*p* < 0.001).

BMI was calculated based on the height and weight of the patients. No statistically significant association was found between BMI and serum vitamin D levels (*p* > 0.05) (Table 2).

**Figure 1:**
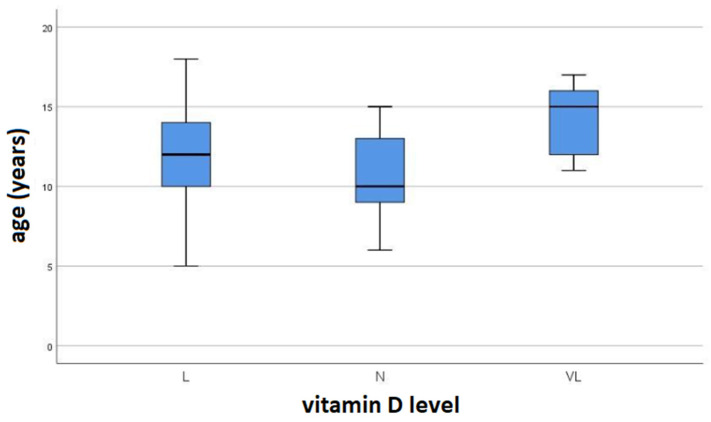
Box and whisker plot for the mean age of patients in relation to serum vitamin D levels (N—normal; L—low; VL—very low vit. D).

Using the ANOVA test, no statistically significant differences in serum vitamin D levels were found based on patient age (*p* > 0.05) (Figure 1).

## 4. Discussion

Fat tissue has a high capacity to store significant amounts of vitamin D [9], especially when adipose tissue volume increases (e.g., in overweight and obesity) [10]. It has been shown that obese individuals have lower circulating levels of the active form of vitamin D compared to those who are not obese, suggesting that the body fat percentage is an important predictor of vitamin D status in different populations [11].

Additionally, vitamin D deficiency can lead to increased calcium release into fat cells due to impaired absorption, where vitamin D plays a crucial role, potentially promoting hyperparathyroidism. Elevated calcium levels in fat cells can stimulate lipogenesis, the process of fat storage. Other studies have shown that high levels of parathyroid hormone (PTH) can increase TAG levels, while higher vitamin D concentrations suppress PTH levels in the serum [12,13]. This suggests that vitamin D may influence TAG concentration by regulating PTH levels. Elevated calcium levels can also enhance the activity of enzymes like fatty acid synthase, which inhibits lipolysis and promotes fat storage [14].

Vitamin D status is also linked to leptin regulation. Leptin is a hormone secreted by adipose tissue that signals satiety to the brain by blocking adipogenesis and stimulating triglyceride hydrolysis [15]. Higher vitamin D levels positively influence leptin levels, promoting lipolysis (fat breakdown) while reducing lipogenesis (fat storage). Some studies suggest that vitamin D plays a functional role in glucose tolerance through its effects on insulin secretion and receptor sensitivity [16]. As a result, low vitamin D levels may contribute to insulin resistance, which is often accompanied by lipid metabolism disorders, increased TAG levels, and decreased HDL levels [17]. Our findings may have clinical relevance for the early identification of children at risk for cardiovascular diseases. Previous research has emphasized the association between vitamin D deficiency and early dyslipidemia, which can contribute to increased cardiovascular risk later in life [18].

In our study, the patients were divided into three groups based on serum vitamin D levels (physiological, low, and very low). These groups were then analyzed for lipid profile parameters (TAG, CHOL, HDL, VLDL, and LDL). The differences in serum vitamin D concentrations were statistically significant (*p* < 0.001). It was determined that there were statistically significant differences regarding TAG, HDL, and VLDL, while for CHOL and LDL, no statistically significant differences were observed.

BMI, which represents the ratio of body mass to height, helps classify patients into different categories to assess whether their weight is within a normal range. In our study, no statistically significant correlation was found between BMI and serum vitamin D levels. However, as vitamin D levels decreased, TAG levels increased, and HDL levels decreased. This finding aligns with a study on pediatric patients with vitamin D deficiency who were not obese but still showed significantly elevated TAG and TAG/HDL ratios, suggesting a potential risk for future dyslipidemia or obesity [3].

A 2022 meta-analysis [19] reported that 22% of pediatric and adolescent populations worldwide exhibit some form of eating disorder. In cases of obesity, expected changes in lipid profiles—higher TAG and LDL levels and lower HDL levels—were observed in a study by Magge S.N. et al. [20]. Regarding vitamin D levels, obesity was associated with vitamin D deficiency, likely due to increased utilization by expanded adipose tissue [21].

Sex and age were also examined in our study. We found no statistically significant differences between serum vitamin D levels and these parameters. A study by Hasan I. et al. [22] similarly reported no significant differences between boys and girls in vitamin D levels, confirming our findings. However, another study by Zhang H. et al. [23] reported lower vitamin D levels in girls and a trend in increasing vitamin D deficiency with age (range: 0–17 years).

According to the German Nutrition Society (Deutsche Gesellschaft für Ernährung, DGE) [24], vitamin D supplementation (10 μg/day) is recommended from the first week of life until the first year and during the winter months of the second year to maintain physiological vitamin D levels. This suggests that in older children, the primary vitamin D source becomes endogenous synthesis through sun exposure, which may explain the tendency for vitamin D deficiency in older pediatric patients.

One limitation of our study is the lack of data on dietary habits, physical activity, and comorbid conditions, which are known to influence both vitamin D status and lipid metabolism. Due to the retrospective design and the nature of data collection from the Laboratory Information System, these variables could not be included in our analysis. Future prospective studies should aim to incorporate these factors to better delineate the independent effect of vitamin D on lipid profiles in children. Additionally, given the cross-sectional design of our study, causality between vitamin D levels and lipid profile alterations cannot be inferred. While our findings highlight a significant association, longitudinal studies are needed to establish a temporal relationship and better understand the potential causal mechanisms underlying the observed dyslipidemia in children with vitamin D deficiency.

## 5. Conclusions

In our study, vitamin D deficiency was accompanied by an increase in TAG and VLDL levels and a decrease in HDL levels. With this in mind, the early detection and identification of abnormal lipid profile parameters in diagnosed vitamin D deficiency could be important for the timely diagnosis of lipid metabolism disorders potentially linked to hypovitaminosis D. This could help prevent widespread non-communicable diseases that arise as a consequence of these associated disorders.

## Figures and Tables

**Table 1:** Lipid status parameters and serum levels of vitamin D.

Parameter (mmol/L)	N (n = 130)	L (n = 114)	VL (n = 88)	*p* *
TAG	0.93 ± 0.12	1.10 ± 0.11	1.13 ± 0.21	0.033 ^a,b,c^
CHOL	4.48 ± 0.26	4.49 ± 0.22	4.53 ± 0.54	ns
HDL	1.30 ± 0.12	1.29 ± 0.09	1.26 ± 0.1	0.042 ^b,c^
LDL	2.76 ± 0.16	2.70 ± 0.17	2.76 ± 0.4	ns
VLDL	0.42 ± 0.07	0.50 ± 0.05	0.51 ± 0.1	0.038 ^a^
TAG:HDL-C	0.72 ± 0.13	0.86 ± 0.11	0.90 ± 0.18	<0.001 ^a,b,c^
Vitamin D	60.11 ± 13.4	39.71 ± 5.36	22.5 ± 4.09	<0.001 ^a,b,c^

The values are mean ± SD. ns—non-significant (>0.05); N—normal; L—low; VL—very low vit. D; TAGs—triacylglycerols (triglycerides); CHOL—cholesterol; HDL—high-density lipoprotein; LDL—low-density lipoprotein; VLDL—very-low-density lipoprotein. * ANCOVA (BMI is a covariate); a—N vs. L; b—N vs. VL; c—L vs. VL.

**Table 2:** Differences in BMI values based on measured serum vitamin D levels.

Parameter	N	L	VL	*p* *
BMI	21.74 ± 6.46	19.6 (17.25–22.25)	19.9 (18.72–27.85)	ns

The values are mean ± SD and median (Q1–Q3). ns—non-significant (>0.05); N—normal; L—low; VL—very low vit. D. * Kruskal–Wallis test.

## Data Availability

Data are available upon reasonable request.

## Abbreviations

The following abbreviations are used in this manuscript:

TAG	Triacylglycerols (triglycerides);
CHOL	Cholesterol;
HDL	High-density lipoprotein;
LDL	Low-density lipoprotein;
VLDL	Very-low-density lipoprotein;
BMI	Body mass index;
CVD	Cardiovascular diseases;
RNI	Reference nutrient intake;
PTH	Parathyroid hormone;
DGE	Deutsche Gesellschaft für Ernährung (German Nutrition Society).

## References

[1] Expert Panel on Integrated Guidelines for Cardiovascular Health and Risk Reduction in Children and Adolescents, National Heart, Lung, and Blood Institute (2011). Expert panel on integrated guidelines for cardiovascular health and risk reduction in children and adolescents: Summary report. Pediatrics.

[2] Jorde R., Figenschau Y., Hutchinson M., Emaus N., Grimnes G. (2010). High serum 25-hydroxyvitamin D concentrations are associated with a favorable serum lipid profile. Eur. J. Clin. Nutr..

[3] Kim M.R., Jeong S.J. (2019). Relationship between vitamin D level and lipid profile in non-obese children. Metabolites.

[4] Holick M.F. (2004). Vitamin D: Importance in the prevention of cancers, type 1 diabetes, heart disease, and osteoporosis. Am. J. Clin. Nutr..

[5] Rajakumar K., Moore C.G., Khalid A.T., Vallejo A.N., Virji M.A., Holick M.F., Greenspan S.L., Arslanian S., Reis S.E. (2020). Effect of vitamin D3 supplementation on vascular and metabolic health of vitamin D-deficient overweight and obese children: A randomized clinical trial. Am. J. Clin. Nutr..

[6] Scientific Advisory Committee on Nutrition (2016). Vitamin D and Health. https://www.gov.uk/government/publications/sacn-vitamin-d-and-health-report.

[7] Radkhah N., Zarezadeh M., Jamilian P., Ostadrahimi A. (2023). The Effect of Vitamin D Supplementation on Lipid Profiles: An Umbrella Review of Meta-Analyses. Adv. Nutr..

[8] (2020). Clinical Practice Guidelines. Vitamin D Deficiency. https://www.rch.org.au/clinicalguide/guideline_index/Vitamin_D_deficiency/.

[9] Al-Oanzi Z.H., Alenazy F.O., Alhassan H.H., Alruwaili Y., Alessa A.I., Alfarm N.B., Alanazi M.O., Alghofaili S.I. (2023). The Role of Vitamin D in Reducing the Risk of Metabolic Disturbances That Cause Cardiovascular Diseases. J. Cardiovasc. Dev. Dis..

[10] Nikparvar M., Khaladeh M., Yousefi H., Vahidi F.M., Moayedi B., Kheirandish M. (2021). Dyslipidemia and its associated factors in southern Iranian women, Bandare-Kong Cohort study, a cross-sectional survey. Sci. Rep..

[11] Dos Santos S.F., Dos Reis Costa P.N., Gouvêa T.G., de Almeida N.F.A., Cardoso F.S. (2023). Influence of hypovitaminosis D during pregnancy on glycemic and lipid profile, inflammatory indicators and anthropometry of pregnant and newborn. Clin. Nutr. ESPEN.

[12] Song S.J., Si S., Liu J., Chen X., Zhou L., Jia G., Liu G., Niu Y., Wu J., Zhang W. (2013). Vitamin D status in Chinese pregnant women and their newborns in Beijing and their relationships to birth size. Public Health Nutr..

[13] Zittermann A., Frisch S., Berthold H.K., Götting C., Kuhn J., Kleesiek K., Stehle P., Koertke H., Koerfer R. (2009). Vitamin D supplementation enhances the beneficial effects of weight loss on cardiovascular disease risk markers. Am. J. Clin. Nutr..

[14] Zemel M.B., Shi H., Greer B., Dirienzo D., Zemel P.C. (2000). Regulation of adiposity by dietary calcium. FASEB J..

[15] Stern J.H., Rutkowski J.M., Scherer P.E. (2016). Adiponectin, Leptin, and Fatty Acids in the Maintenance of Metabolic Homeostasis through Adipose Tissue Crosstalk. Cell Metab..

[16] Palomer X., Gonzalez-Clemente J.M., Blanco-Vaca F., Mauricio D. (2008). Role of vitamin D in the pathogenesis of type 2 diabetes mellitus. Diabetes Obes. Metab..

[17] Chiu K.C., Chu A., Go V.L., Saad M.F. (2004). Hypovitaminosis D is associated with insulin resistance and beta cell dysfunction. Am. J. Clin. Nutr..

[18] Sodero G., Rigante D., Pane L.C., Sessa L., Quarta L., Candelli M., Cipolla C. (2024). Cardiometabolic Risk Assessment in a Cohort of Children and Adolescents Diagnosed with Hyperinsulinemia. Diseases.

[19] López-Gil J.F., García-Hermoso A., Smith L., Firth J., Trott M., Mesas A.E., Jiménez-López E., Gutiérrez-Espinoza H., Tárraga-López P.J., Victoria-Montesinos D. (2023). Global Proportion of Disordered Eating in Children and Adolescents: A Systematic Review and Meta-analysis. JAMA Pediatr..

[20] Magge S.N., Goodman E., Armstrong S.C., Committee on Nutrition, Section on Endocrinology, Section on Obesity (2017). The Metabolic Syndrome in Children and Adolescents: Shifting the Focus to Cardiometabolic Risk Factor Clustering. Pediatrics.

[21] Migliaccio S., Di Nisio A., Mele C., Scappaticcio L., Savastano S., Colao A., Obesity Programs of nutrition, Education, Research and Assessment (OPERA) Group (2019). Obesity and hypovitaminosis D: Causality or casualty?. Int. J. Obes. Suppl..

[22] Isa H., Almaliki M., Alsabea A., Mohamed A. (2020). Vitamin D deficiency in healthy children in Bahrain: Do gender and age matter?. East. Mediterr. Health J..

[23] Zhang H., Li Z., Wei Y., Fu J., Feng Y., Chen D., Xu D. (2020). Status and influential factors of vitamin D among children aged 0 to 6 years in a Chinese population. BMC Public Health.

[24] German Nutrition Society (2012). New reference values for vitamin D. Ann. Nutr. Metab..

